# High baseball loads induce shoulder and elbow injuries among high school baseball pitchers: a prospective study

**DOI:** 10.1038/s41598-020-79841-7

**Published:** 2021-01-11

**Authors:** Hitoshi Shitara, Tsuyoshi Tajika, Takuro Kuboi, Tsuyoshi Ichinose, Tsuyoshi Sasaki, Noritaka Hamano, Takafumi Endo, Masataka Kamiyama, Daisuke Simoyama, Junki Suzuki, Atsushi Yamamoto, Tsutomu Kobayashi, Kenji Takagishi, Hirotaka Chikuda

**Affiliations:** Department of Orthopaedic Surgery, Gunma University Graduate School of Medicine, 3-39-22, Showa, Maebashi, Gunma 371-8511 Japan

**Keywords:** Health care, Medical research, Risk factors

## Abstract

Studies on the relationship between baseball loads (practice, training, and competition hours) and shoulder and elbow injuries among high school pitchers are limited. Therefore, this study included 92 male high school baseball pitchers and evaluated their preseason shoulder and elbow conditions. All participants completed a self-recorded questionnaire regarding baseball load, presence of shoulder pain or elbow pain, or both, and pitching limitations due to shoulder and/or elbow pain experienced daily to determine the occurrence of injuries and record the baseball load. The optimal load cutoff value was determined using a receiver operating characteristic curve analysis. Participants were categorized into high-load and low-load groups according to the aforementioned cutoff value. The Kaplan–Meier method was used to obtain time-to-event curves, and cox proportional hazards models were used to calculate the hazard ratios for injury rates. The cutoff value of the average baseball load was 324.4 min per day. A high load (> 5.5 h/day) led to a 2.6-times greater risk of injuries and 3.3-times earlier occurrence of injuries than a low load (< 5.5 h/day). Therefore, a high load is a risk factor for shoulder and elbow injuries in high school baseball pitchers.

## Introduction

Shoulder and elbow injuries and pain are major issues for baseball players^1–3^. Although many studies have demonstrated the risk factors for baseball-related shoulder and elbow injuries, a recent systematic review^4^ showed that there have been only 14 prospective cohort studies: 8 studies involving professional players (major league and minor league), 4 studies involving high school players, and 2 studies involving youth players.

Among professional baseball players, shoulder external rotation and elbow varus torque at peak external shoulder rotation during pitching^5^, high pitch velocity^6^, glenohumeral internal rotation deficit (GIRD), shoulder external rotation insufficiency^7^, total shoulder rotation deficit during the preseason, deficits in the supraspinatus during the preseason, and prone external rotation (PER) strength^8^ are risk factors for shoulder and elbow injuries. Shitara et al.^9^ showed that, among high school baseball players, GIRD and greater differences in prone external rotation strength of the arms increased the risk for shoulder and elbow injuries. Tyler et al.^10^ showed that shoulder and elbow injury risks were not increased for pitchers who experienced an excessive loss of internal range of motion (ROM) or loss of total shoulder ROM, and that only supraspinatus weakness during the preseason was significantly associated with major injuries. Among youth baseball players, significant risk factors for elbow injuries were a history of elbow pain^11^, age 9–11 years^11^, playing the position of pitcher or catcher^11^, pitching more than 100 innings in a year^12^, and training more than 16 h per week^11^.

As described, evidence of preseason risk factors, such as ROM and strength measured during the preseason, is relatively sufficient; however, evidence of in-season risk factors, such as the number of innings played and external load (practice, training and competition hours), is limited. In particular, there is no evidence of the relationship between the baseball load duration and the incidence of shoulder and elbow injuries among high school baseball pitchers.

For adolescent pitchers, risk factors are related to the number of innings pitched in the games and the duration of practice. The training duration affects adolescent pitchers more than established baseball pitchers, such as college and professional pitchers. This may be because adolescent baseball pitchers spend more time with non-pitchers and engage more in daily practices, including warming up, batting, pitching, defensive drilling, cooling down, and strength workouts, than practice for pitcher-specific exercises. Additionally, measuring the duration of the baseball load is easier than counting the number of pitches or innings. If pitchers’ own measurement of a high baseball load is related to shoulder and elbow injuries, a simple self-assessment of the total duration of practice, training, and game time will contribute to preventing injuries.

We hypothesized that the high amount of baseball load (training and competition hours) in adolescent baseball pitchers induced shoulder and elbow injuries. To test the hypothesis, we prospectively investigated the relationship between the baseball load, defined as the total duration of daily team practice, including warming up, batting, pitching, defensive drills, cooling down, strength workouts, and games of high school baseball pitchers and the incidence of shoulder and elbow injuries.

## Results

### Receiver operating characteristics (ROC) analysis

**Figure 1:**
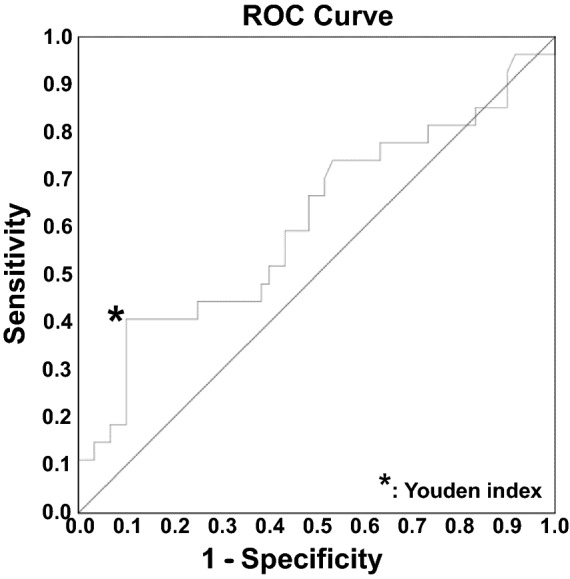
Receiver operating characteristics (ROC) curve. The ROC analysis shows that the cutoff value of the average baseball load is 324.4 min per day (P = 0.10, area under the curve [AOC] = 0.60; Fig. 1).

The ROC analysis showed that the cutoff value of the average baseball load was 324.4 min per day (P = 0.10; area under the curve (AUC) = 0.60, Fig. 1).

### Baseline characteristics

The average duration of games and practice per week were 452 and 1259 min (7 h 32 min and 20 h 59 min), respectively. Similarly, the average duration of games and practice per day were 395 and 260 min (6 h 35 min and 4 h 46 min), respectively. Based on the cutoff value, participants were categorized into the high baseball load group (high group) and the low baseball load group (low group). There were 16 and 76 pitchers in the high group and low group, respectively. The preseason baseline assessment indicated no significant difference between both groups regarding baseball experience, height, weight, ROM of ABIR and ROM of horizontal adduction (HA) in the dominant shoulder, elbow flexion and extension on the dominant side, PER and prone internal rotation (PIR) in the dominant shoulder, and PER and PIR ratios (Table 1). Thus, according to these preseason measurements, participants in the two groups had the same risk of experiencing shoulder and elbow injuries during the season.

**Table 1:** Baseline characteristics of the study participants.

Baseline characteristics	Low group (N = 76)		High group (N = 16)		*P value*
	Mean	SD	Mean	SD	
Baseball experience (years)	8.1	1.9	8.7	2.0	0.32
Body height (cm)	172.8	5.2	173.4	5.1	0.71
Body weight (kg)	67.9	6.6	68.7	6.2	0.64
ABIR on the dominant side (deg)	36.0	13.6	42.1	12.6	0.10
HA on the dominant side (deg)	27.9	13.3	32.1	6.4	0.22
Elbow flexion on the dominant side (deg)	144.0	4.8	143.4	5.4	0.66
Elbow extension on the dominant side (deg)	3.0	5.8	0.6	9.9	0.19
PER on dominant side (lb)	23.8	5.5	25.3	6.5	0.36
PER ratio	1.0	0.2	0.9	0.1	0.54
PIR on the dominant side (lb)	25.8	7.6	27.1	9.8	0.28
PIR ratio	1.0	0.2	0.9	0.2	0.48

*ABIR* ROM of 90° of abducted internal rotation in the shoulder, *HA* ROM of horizontal adduction in the shoulder, *PER* prone external rotation, *PIR* prone internal rotation, *ratio* strength of the dominant side/strength of the nondominant side, *SD* standard deviation, *ABIR* abducted internal rotation, *HA* horizontal adduction, *ROM* range of motion.

### Time-to-event analysis

**Figure 2:**
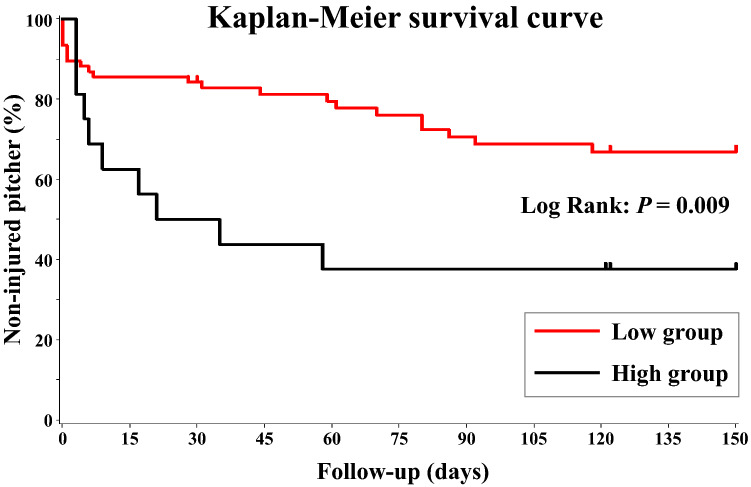
Kaplan–Meier survival curves. The median time to injury was 92 days and 28 days in the low group and high group, respectively. A log-rank test shows that the injury incidence is significantly lower in the low group than in the high group (P = 0.009).

The injury rate of the high group was 62.5% (n = 10); however, it was 28.9% (n = 22) for the low group. The median time to injury was 92 and 28 days for the low and high groups, respectively (Fig. 2). This suggested that a high baseball load led to a 3.3-times earlier occurrence of injuries. The Kaplan–Meier analysis yielded a hazard ratio (HR) of 2.603 for the high group (Table 2). A log-rank test showed that the injury incidence was significantly lower in the low group than in the high group (P = 0.009) (Fig. 2). Additionally, injuries occurred 3.3-times earlier in the high group than in the low group.

**Table 2:** Results of the Cox proportional hazards model.

Group	Total	Injury occurrence	
	N	N (%)	HR (95% CI)
Low group	76	22 (28.9)	1
High group	16	10 (62.5)	2.603 (1.229–5.517)

*HR* hazard ratio, *CI* confidence interval.

### Post hoc power analysis

A post hoc power analysis showed that the statistical power of this study was 0.831.

## Discussion

The most important finding of this study was that a high baseball load (> 5.5 h per day) for high school baseball pitchers significantly increased the risk of shoulder and elbow injuries. Furthermore, our results demonstrated that a high baseball load led to a 2.6-fold greater risk of injuries and 3.3-times earlier occurrence of injuries. No previous prospective study of high school pitchers has provided evidence that an excessively high baseball load induces shoulder and elbow injuries. Therefore, this evidence may help determine the appropriate baseball load necessary to prevent shoulder and elbow injuries in youth pitchers.

### Training and competition hours as an external load

A baseball load of more than 5 h a day is considered high, exceeding the common load in the United States. However, adolescent Japanese baseball players often perform baseball practice not only after school but also in the morning before school classes begin; thus, the baseball load sometimes exceeds 5 h.

Takagishi et al.^3^ performed a retrospective nationwide survey of junior high school baseball players and demonstrated that there was no significant relationship between the total number of hours of practice per week, the total number of days of practice per week, and the presence of shoulder pain and elbow pain. Only one previous prospective study demonstrated that training more than 16 h per week^11^ is a significant risk factor for shoulder pain and elbow pain in youth players.

Matsuura et al.^11^ reported that youth baseball players who trained more than 16 h but 36 h or less per week were at significantly high risk for shoulder pain and elbow pain compared to those who trained 10 h or less per week (odds ratio = 2.00 and odds ratio = 2.33, respectively). This evidence is important for protecting young baseball players from preventable shoulder and elbow injuries. However, unfortunately, there were several limitations to the study by Matsuura et al.^11^. First, baseline conditions, such as GRID, total ROM in the shoulder, muscle strength, and scapular dyskinesia, were unclear in the pain and non-pain groups because physical examinations were not performed during the study. Second, there might have been recall bias due to vague memory because young participants, including 6-year-old players, were asked to recount their history of shoulder pain and/or elbow pain. Third, in that study, pain was defined as “present” if it restricted participation in baseball for 1 day or more; this definition seemed too restrictive for a study investigating risk. It is difficult to directly compare the present study with that by Shitara et al.^9^ because the definitions of shoulder and elbow injury events are different; our cutoff value of the average baseball load was calculated as 37.8 h per week (324.4 min × 7 days), and our baseball load for high school pitchers was longer than theirs for youth baseball players. In this study, we performed physical examinations to confirm no significant difference between the baseline conditions of the high group and low group. Additionally, the participants were asked to record their baseball load daily to avoid recall bias. Finally, our definition of “shoulder or elbow injury” was any condition resulting in the pitcher being considered disabled for 8 days or more, which was applied in previous studies^9,13,14^ and, therefore, might be more meaningful for investigating risk factors. By addressing the issues of the previous study, we demonstrated that a high baseball load (> 5.5 h) led to a 2.6-times greater risk of injuries and 3.3-fold earlier occurrence of injuries compared to a low baseball load (< 5.5 h), even when there were no significant differences in the baseline characteristics of the groups.

### Physical findings and external load

Møller et al.^15^ investigated the relationship between shoulder injury and a handball load (training and competition hours) of more than 31 weeks for handball players (age, 14–18 years); they reported that a 60% increase in the handball load could increase the shoulder injury rate, even for players with normal shoulder characteristics. Moreover, they reported that, compared to players with normal scapular control and external rotational strength, players with scapular dyskinesis and reduced external rotational strength during the preseason were predisposed to shoulder injury when the handball load was moderately increased^15^. These findings suggest that reduced external rotational strength or scapular dyskinesis enhanced the effect of the external load on the injury rate. Because reduced external rotational strength among high school pitchers^9^ and professional pitchers^8^ and scapular dyskinesis among collegiate baseball players^16^ are known risk factors for shoulder and elbow injuries, excessive external load during the season might exacerbate scapular function. Therefore, external rotation of the shoulder might induce injuries. Further studies are needed to clarify these relationships.

### Limitations

This study had some limitations. First, data regarding other external load factors, such as the total number of pitches and the number of innings pitched, were not collected during this study. These factors might be correlated with training and competition hours and could be confounding. Although it is a study limitation, baseball load is a more comprehensive factor to consider for its contribution to baseball practice and games, rather than the number of pitches or innings. Therefore, we measured the baseball load instead of the number of pitches or innings. Furthermore, all practices and game plans of the participants were mainly managed by the team instead of individuals because all high school baseball players in Japan belong to their school’s teams. Therefore, participants spend almost equal time doing baseball practice and training as a team activity (which we collected in this study) as they do individual physical training or baseball practice. However, it is a limitation that we did not collect the details of the baseball load.

Additionally, it is difficult to apply the evidence in this study to baseball players in other countries because the environment surrounding baseball is different across countries. Therefore, this should be considered carefully in future studies. Second, the sample size was relatively small because the incidence of shoulder and elbow injuries was relatively low for high school baseball pitchers. However, the post hoc power analysis showed that the statistical power of the study was 0.831, indicating that the sample size was sufficient for testing the relationship between the external load and shoulder and elbow injuries. Third, it remains unclear how the increased baseball load induced a high injury rate because we did not perform a physical examination or imaging study when the injury occurred. Fourth, there is a possibility for selection bias because we excluded pitchers performing muscle-specific rotator cuff exercises or posterior capsular stretching exercises outside of those performed during team exercises. Although we aimed to reduce the bias by selecting interventions, which were reported to reduce shoulder and elbow injuries among high school baseball pitchers, the selected participants may be at higher risk for these injuries. However, we believe that the effect was minimal because we continuously recommended all teams to perform general physical conditioning exercises, such as whole-body stretching and strength training, as a team exercise, and no pitcher was excluded for this criterion. Finally, the relationship between the severity of injuries and the baseball load is unclear because we did not collect data regarding injury severity.

### Conclusion

For high school baseball pitchers, a high baseball load (> 5.5 h/day) led to a 2.6-times greater risk of injuries and 3.3-times earlier occurrence of injuries compared to a low baseball load (< 5.5 h/day). Although this evidence requires validation in future studies, it may help to provide guidelines for preventing injuries among youth baseball pitchers. These results can be used to establish the basis for discussing the appropriate baseball load to prevent injuries because the evidence indicated that a high baseball load is a risk factor for shoulder and elbow injuries among high school baseball pitchers.

## Methods

### Participants

Preseason medical examinations were performed for 131 high school male baseball pitchers between the ages of 15 and 17 years. Subsequently, informed consent was obtained from the parents of the participants. Finally, 92 pitchers were enrolled in this study.

Following the inclusion criteria of a previous study^9^, a pitcher was included in our study if he participated in preseason workouts as an active pitcher and experienced no pitching activity restrictions. In contrast, the exclusion criteria were as follows: prior injuries (e.g., fracture) of the throwing arm; inability to throw or restricted pitching activity because of a shoulder or elbow problem; or involvement in daily muscle-specific rotator cuff training exercises or posterior capsule/sleeper stretches other than those performed during team exercises. The Institutional Review Board of Gunma University Hospital (identification number 1003) approved this study. All methods were performed in accordance with relevant guidelines and regulations.

### Medical examinations

As previously reported^9,13^, preseason baseline medical examinations were performed to evaluate the preseason condition of the participants’ shoulders and elbows. The hand dominance of the participants was unknown to the examiners. Baseball experience, height, weight, shoulder ROM, elbow ROM, and shoulder muscle strength were evaluated.

#### ROM measurements

As previously reported^9,13^, shoulder ROM of 90° of abducted internal rotation (ABIR) and HA and elbow ROM of flexion and extension were measured by a certified orthopedic surgeon using a digital protractor (iGaging, Los Angeles, CA, USA).

The intra-rater validity and reliability of the ROM measurements using a digital protractor have been established by a previous study^9^. Passive ROM of ABIR was measured in the supine position with stabilization of the scapula by applying a posterior force to the coracoid process. When measuring ABIR, a digital protractor was placed on the forearm. Passive ROM of HA was measured in the supine position with stabilization of the axillary border of the scapula. When measuring HA, a digital protractor was placed on the humerus. Similarly, passive ROM of elbow flexion and passive ROM of the extension were measured in the supine position.

#### Strength measurements

PIR strength and PER strength of the shoulder were measured using a PowerTrack II Commander hand-held dynamometer (J-Tech Medical, Salt Lake City, UT, USA) by a certified orthopedic surgeon, as previously reported^8,9,13^. The intra-rater validity and reliability of hand-held dynamometers have been established in a previous study^9^. The median value of the three repetition trials was recorded and subsequently analyzed. PER was measured in the prone position with the humerus in 90° of abduction and the elbow in 90° of flexion. The arm was set in a neutral position, and the examiner stabilized the humerus. Participants were asked to rotate the arm externally with maximum power against the dynamometer placed on the dorsal side of the forearm, 5 cm proximal to the proximal wrist extension crease. PIR was measured similarly, except that the dynamometer was placed on the volar side of the forearm, 5 cm proximal to the proximal wrist flexion crease. Similarly, participants were asked to internally rotate the arm with maximum power. The dominant-to-nondominant ratios of PER and PIR were calculated for each participant and analyzed.

### Injury tracking and season data collection

Shoulder- and elbow injuries were defined as any condition resulting in the pitcher being considered disabled for 8 days or more^9,13,14^. Other injuries that occurred via other mechanisms, such as trauma from falls, collisions with other players, sprains while running, or being hit by a pitch, were not included in the statistical analyses. To detect when injuries occurred and record the exact baseball load, participants were asked to complete a self-recorded questionnaire regarding baseball load, the presence of shoulder pain or elbow pain, or both, and limitations to pitching caused by shoulder pain or elbow pain. To avoid recall bias, participants completed the questionnaire daily and returned the record to us monthly. Furthermore, we called the participants once or twice each month to verify that they were completing the daily questionnaires.

### Statistical analysis

We performed statistical analyses using SAS 9.4 (SAS Institute Inc., Cary, NC, USA). All tests were two-sided with a significance level of P ≤ 0.05. A receiver operating characteristic (ROC) curve analysis was performed to detect the cutoff value of the baseball load. Subsequently, participants were categorized into two groups using the detected cutoff value based on the Youden index. The Mann–Whitney U test was used to evaluate group differences. The Kaplan–Meier method was used to obtain time-to-event curves, and HRs for the incidence of injury were calculated using Cox proportional hazards models. A log-rank test was performed to compare the survival distributions between groups.

To calculate the required number of participants, we performed an a priori statistical power analysis, which indicated that we needed 39 participants to achieve a statistical power of 80% at an α level of 0.05 with an HR of 2.7^9^, an accrual interval of 150 days, a follow-up interval of 150 days, and a median time to failure for each group; the shortest time to failure was 50 days according to the Kaplan–Meier analysis^17^. After data collection, we performed a post hoc power analysis to evaluate the statistical power of this study.

## Data Availability

The data supporting the findings of this study are available on request from the corresponding author H.S. The data are not publicly available because they contain information that could compromise the privacy of research participants.
